# Identifying challenges in neurofibromatosis: a modified Delphi procedure

**DOI:** 10.1038/s41431-021-00892-z

**Published:** 2021-04-26

**Authors:** Britt A. E. Dhaenens, Rosalie E. Ferner, Annette Bakker, Marco Nievo, D. Gareth Evans, Pierre Wolkenstein, Cornelia Potratz, Scott R. Plotkin, Guenter Heimann, Eric Legius, Rianne Oostenbrink

**Affiliations:** 1 Department of General Paediatrics, Sophia’s Children’s Hospital, Rotterdam, The Netherlands; 2 ENCORE, Erasmus MC, Rotterdam, The Netherlands; 3 Department of Neurology, Guy’s and St. Thomas’ NHS Foundation Trust, London, UK; 4 Children’s Tumor Foundation, New York, NY USA; 5 Centre for Genomic Medicine, Division of Evolution and Genomic Sciences, University of Manchester, St Mary’s Hospital, Manchester, UK; 6 Department of Dermatology, Hôpital Universitaire Pitié-Salpêtrière (APHP), Paris, France; 7 Department of Paediatric Neurology, Charité Universitätsmedizin Berlin, Berlin, Germany; 8 Department of Neurology and Cancer Center, Massachusetts General Hospital, Boston, MA USA; 9 Biostatistics & Pharmacometrics, Novartis Pharma AG, Basel, Switzerland; 10 Department of Clinical Genetics, UZ Leuven, Leuven, Belgium

**Keywords:** Neurodevelopmental disorders, Adaptive clinical trial

## Abstract

Neurofibromatosis type 1 (NF1), neurofibromatosis type 2 (NF2) and schwannomatosis (SWN) are rare conditions with pronounced variability of clinical expression. We aimed to reach consensus on the most important manifestations meriting the development of drug trials. The five-staged modified Delphi procedure consisted of two questionnaires and a consensus meeting for 40 NF experts, a survey for 63 patient representatives, and a final workshop. In the questionnaires, manifestations were scored on multiple items on a 4-point Likert scale. The highest average scores for NF experts deciding the ‘need for new treatment’ were for malignant peripheral nerve sheath tumour (MPNST) (4,0) and high grade glioma (HGG) (3,9) for NF1; meningioma (3,9) for NF2 and pain (3,9) for SWN. The patient representatives assigned high scores to all manifestations, with plexiform neurofibroma being highest in NF1 (4,0), vestibular schwannoma in NF2 (4,0), and pain in SWN (3,9). Twelve experts participated in the consensus meeting and prioritised manifestations. MPNST was ranked the highest for NF1, followed by benign peripheral nerve sheath tumours. Tumour manifestations received highest ranking in NF2, and pain was the most prominent problem for SWN. Patient representative ratings for NF1 were similar to the experts’ opinions, except that they ranked HGG as the most important manifestation. For NF2 and SWN, the patient representatives agreed with the experts. We conclude that NF experts and patient representatives consent to prioritise development of drug trials for MPNST, benign peripheral nerve sheath tumours, cutaneous manifestations and HGG for NF1; tumours for NF2; and pain for SWN.

## Introduction

Neurofibromatosis type 1 (NF1), neurofibromatosis type 2 (NF2) and schwannomatosis (SWN) are genetic disorders that predispose to the development of nerve sheath tumours [1, 2]. These tumours are mostly benign with a low chance of malignant transformation but can cause significant neurological morbidity due to their size and/or location. All three diseases are autosomal dominantly inherited, with a high de novo mutation rate (50% in NF1 and NF2) and characterised by a prominent variability in expression [1, 3, 4].

Rare hereditary conditions like the neurofibromatoses (NF) require large, multicentre trials and multiple patient populations for successful evaluation of new treatments. EU-PEARL (EU Patient-Centric Clinical Trial Platforms) is an international project, and the aim is to create a framework for the future conduct of Integrated Research Platforms (IRPs) [5]. Instead of conducting multiple single clinical drug trials, the goal of IRPs is to accelerate the development of new treatments and to reduce operational costs, something that is much needed in NF and health care in general.

The wide range of manifestations of NF (especially in NF1) presents a challenge when trying to create a framework for future IRPs. Since it would be impossible to include all disease manifestations, it is critical to prioritise clinical manifestations for evaluation. Given the patient-centric design of EU-PEARL and the general importance of including patients’ opinion in clinical trial design, patient input on this prioritisation is vital. The aim of this study was to reach consensus on the most important manifestations of NF to select for clinical drug trials, based on the opinions of both NF experts and patient representatives.

## Methods

**Fig. 1:**
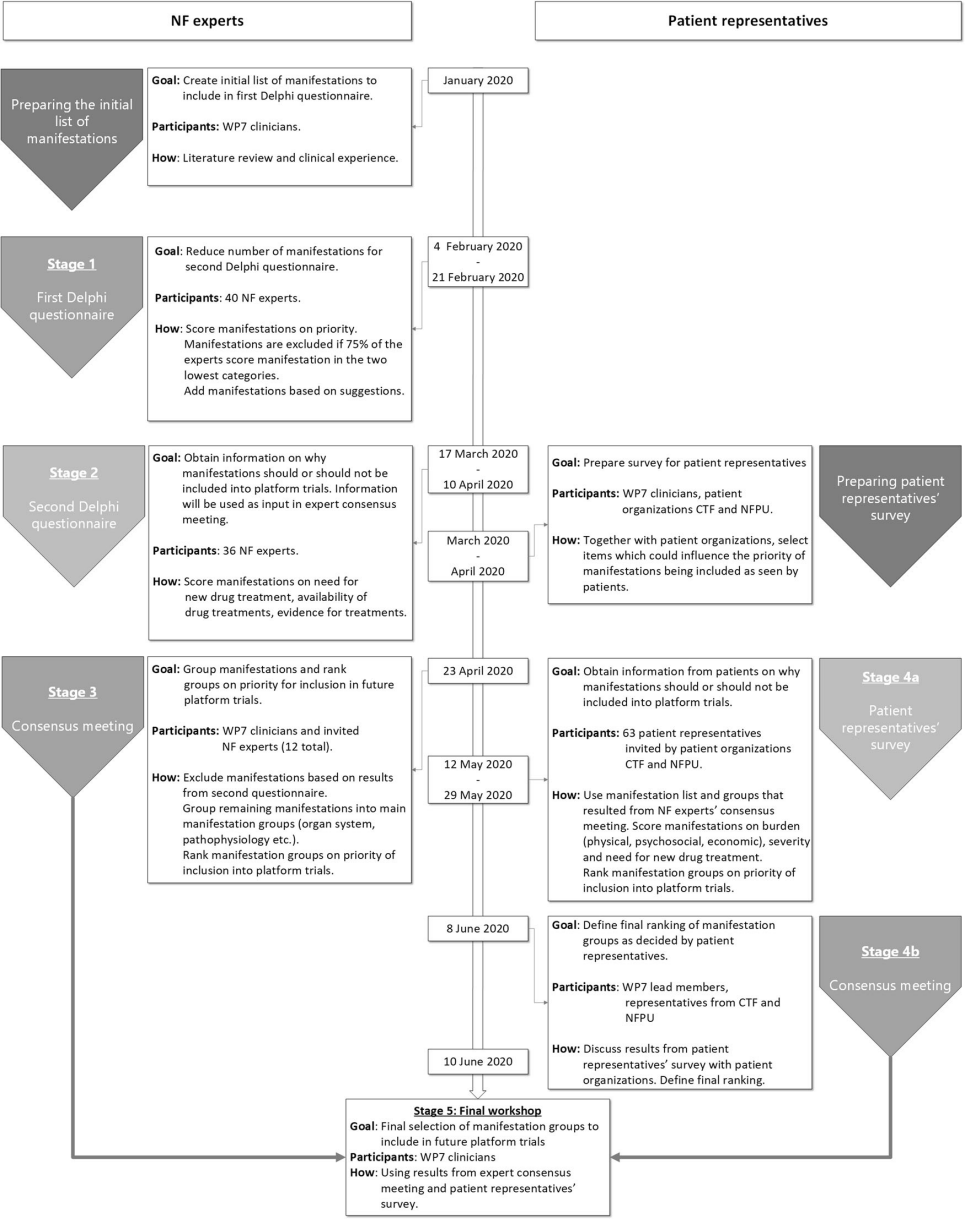
A flowchart depicting the multiple stages of the study. The study consisted of two pathways, one for NF experts and one for patient representatives. The expert pathway consisted of two Delphi questionnaires and a consensus meeting, the patient representatives had one survey and a consensus meeting. The final selection of manifestations was done in a final workshop. *CTF* Children’s Tumour Foundation, *NFPU* Neurofibromatosis Patients United, WP7 clinicians clinicians involved in work package 7 of the EU-PEARL project, dedicated to neurofibromatosis.

We used a five-staged modified Delphi procedure, consisting of two questionnaires and a consensus meeting for NF experts, a survey and consensus meeting for patient representatives, and a final workshop for the selection of manifestations (Fig. 1).

### Preparation of the initial list of manifestations

Initially, we prepared the list of clinical manifestations that would be presented to the NF experts in the first questionnaire. Based on a literature search in Medline [6, 7] and the clinical experience within our work package group of EU-PEARL (work package 7 (WP7)), we produced a list of the most common and important manifestations for NF1, NF2 and SWN to be included in the first questionnaire to NF experts.

### Selecting and contacting NF experts for the Delphi questionnaires and consensus meeting

Potential Delphi participants were included from our contacts through (a) the European Neurofibromatosis Group (ENFG), (b) NF experts within ERN GENTURIS, (c) clinicians from the Children’s Tumour Foundation (CTF) clinical care advisory board and (d) international NF experts who participated in the development of the new diagnostic criteria [8]. We did not use a set definition of ‘an expert’, as this is arbitrary. There is no known method to calculate the needed group size for a Delphi procedure [9], since it is often researcher and situation specific [10]. We estimated that 40–50 NF experts would be a convenient sample size for the Delphi. Fifty-two possible participants were invited by email to participate in the Delphi and informed of the estimated time required to complete the questionnaires. Positive responders to this email were included into both Delphi questionnaires. A subset of these positive responders (*n* = 12) were asked to participate in the NF expert consensus meeting.

At the start of each questionnaire participants received an announcement email, followed by a second email with a hyperlink to the questionnaire. The deadline for completing the questionnaires was set at 2, 5 weeks after sending the hyperlink. Non-responders were sent a general reminder after two weeks, and a personalised reminder email on the day of the deadline. The questionnaires were built and distributed using Google Forms.

### Stage 1: First Delphi questionnaire for NF experts

The first questionnaire aimed to reduce the number of manifestations. Participants were asked to score each manifestation on a 4-point Likert scale (1 = ‘No priority’ to 4 = ‘High priority’) for priority of inclusion into a platform trial (ANNEX 1). Manifestations were excluded from the second Delphi questionnaire if ≥75% of the participants rated the manifestation as low priority or no priority (score ≤ 2).

### Stage 2: Second Delphi questionnaire for NF experts

The second questionnaire aimed to obtain information on why manifestations should or should not be included into a platform trial. Participants were asked to rate the manifestations on: (i) the need for a new drug treatment in addition to existing treatments, (ii) the availability of existing drug treatments and (iii) the available evidence for these treatments. Need for new treatments and availability of drug therapies were scored on a 4-point Likert scale, and evidence for effectiveness of existing drug therapies on a 5-point Likert scale. For items (ii) availability and (iii) evidence, a ‘Do not know’ option was provided. Answers from experts who chose this option were excluded from the analysis for that item.

### Stage 3: Consensus meeting for NF experts

The consensus meeting was hosted virtually due to the COVID-19 pandemic and planned two weeks after the deadline of the second Delphi questionnaire. The consensus meeting had two goals: (i) to arrange the various NF manifestations into manifestation groups that could be studied in a combined platform trial, and (ii) to reach consensus about a priority ranking for these groups. First, manifestations were excluded in a group discussion, based on the results from the second questionnaire and clinical expertise of participants. Next, the remaining manifestations were aggregated into groups, based on pathophysiology, targets for treatments, organ system, etc. Participants were asked to rank these manifestation groups according to priority for inclusion into platform trials. Given the larger number of manifestations for NF1, this condition required a third questionnaire. It consisted of a question on feasibility of performing a platform trial for this group (easy or difficult) and the ranking of the manifestation groups.

### Stage 4a and 4b: Patient representatives’ survey and consensus meeting

To include input from patients in our final selection of manifestations, a survey and consensus meeting was performed for patient representatives. Separate surveys were developed for NF1, NF2 and SWN patient representatives in close coordination with two NF patient organisations (Neurofibromatosis Patients United (NFPU) and CTF) [11, 12]. Together with a group of patient representatives selected from these organisations, two WP7 members (BD, RO) identified five items that could influence patient representatives’ priority for inclusion of manifestations: burden (physical, psychosocial and economic burden), severity and the need for new drug treatments. Patient representatives were recruited through the patient organisations: they were eligible if they were able to read and answer the survey in the English language. Patient representatives could be patients themselves, parents of patients, or unaffected individuals who were closely involved in NF patient organisations. An invitation email with the link to the surveys was sent directly to known patient representatives or to other patient organisations, with the request to forward the survey to their patient representatives. The deadline for completing the surveys was set at two weeks after launch. General reminder emails were sent 1 week and 1, 5 weeks after launch. Participants completed the survey solely for the condition that they represented (NF1 patient representatives answered the NF1 survey, NF2 and SWN representatives answered the NF2 and SWN respectively). Participants were asked to score the five previously mentioned items on a 4-point Likert scale. This was done for the manifestation list that resulted from the NF expert consensus meeting. They were also asked to rank the groups of manifestations according to priority for finding new treatments. The patient representatives did not know the results of the ranking from the NF expert consensus meeting. Results were discussed among patient representatives from NFPU and CTF and WP7 members (BD, AB, MN) in a consensus meeting hosted virtually, and a consensus was reached on the final ranking of manifestation groups as seen by patient representatives.

### Stage 5: Final workshop

The outcomes of the NF expert consensus meeting and the patient representatives’ survey and consensus meeting were used by the WP7 group to decide on a final selection of manifestations groups in a final virtual workshop.

### Data analysis

Results from the second Delphi questionnaire for NF experts and the patient representatives’ survey were analysed by calculating the average score on the Likert scale for each item. Additionally, all items were analysed for floor and ceiling effects (75% of the respondents allotted either the highest or the lowest score). For the NF expert consensus meeting and patient representatives’ survey, average rankings were calculated. The ranking could range between 1 and 8 for NF1 (since eight manifestation groups could be ranked); between 1 and 2 for NF2, and between 1 and 3 for SWN. A lower ranking implies a higher priority for inclusion into future clinical trials.

## Results

We identified a total of 66 manifestations; 52 for NF1, 9 for NF2 and 5 for SWN.

### Stage 1: First Delphi questionnaire for NF experts

There were 43 positive responses to the Delphi invitation and 9 individuals did not respond. Thirty-eight participants completed the first questionnaire in the given timeframe (Fig. 1). The questionnaire was reopened temporarily for two days at the request of two additional respondents, achieving 40 respondents in total (93%). Thirty-two out of these 40 respondents reported expertise in NF1 (80%), 24 in NF2 (60%) and 17 in SWN (43%). Fourteen NF1 manifestations were excluded using the pre-defined criteria. After reviewing the suggestions for additional manifestations and rephrasing, one manifestation was added for both NF1 (problems with motor skills and/or coordination) and NF2 (mononeuropathy). Four manifestations were rephrased, and for NF1, ‘brain or spinal cord glioma’ was split into three separate manifestations: low grade brain glioma, high grade brain glioma, and low grade spinal cord glioma. A total of 55 manifestations remained; 40 for NF1, 10 for NF2 and 5 for SWN (ANNEX 2).

### Stage 2: Second Delphi questionnaire for NF experts

Due to the COVID-19 outbreak the original deadline of 2, 5 weeks after launch was extended by 1 week. Thirty-six participants completed the questionnaire (84%) (Fig. 1). For NF1, the manifestations with the highest average score for need for treatment (NT) were the malignant peripheral nerve sheath tumour (MPNST) (4,0), high grade glioma (HGG) (3,9) and plexiform neurofibroma (3,8) (ANNEX 3). Seven manifestations displayed a ceiling effect: (sub)cutaneous neurofibroma, plexiform neurofibroma, atypical neurofibroma, spinal nerve root neurofibroma, high grade glioma and MPNST. No floor effects were observed in NT scores. In NF2 the highest score for NT was observed in meningioma (3,9) (ANNEX 4). The visual complications cataract (2,2) and retinal hamartoma (2,4) were appointed the lowest average NT scores. Vestibular schwannoma and meningioma displayed a ceiling effect. Pain received the highest average NT score for schwannomatosis (3,9) (ANNEX 4) and was also the only manifestation to display a ceiling effect. The other manifestations obtained high average scores but showed higher variability, as reflected in the distribution of appointed scores (ANNEX 4).

### Stage 3: Consensus meeting for NF experts

**Fig. 2:**
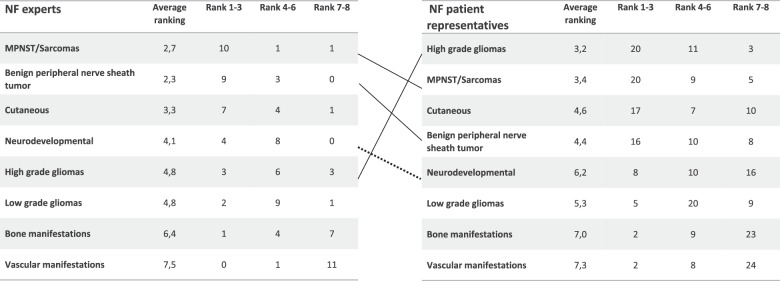
Distribution of the rankings of NF1 manifestation groups as given during the expert consensus meeting and the patient representatives’ survey. A lower ranking means higher priority for inclusion in clinical trials. Manifestation groups are sorted to the number of votes in the highest priority rankings (rank 1–3). Dotted lines: manifestation group not included in final selection.

Seven external NF experts and five WP7 clinicians participated in the consensus meeting. In the NF1 consensus discussion, a number of manifestations were excluded based on the availability of existing effective treatments (e.g. vitamin D deficiency, precocious puberty, growth hormone deficiency), which was reflected in their average scores for availability and evidence for treatment (ANNEX 5). Two manifestations were excluded due to limited feasibility to perform trials (fatigue/strength, sleep disorder). The remaining manifestations were grouped into 10 main groups (ANNEX 3). It was decided not to include the groups ‘Other malignancies’ and ‘Other manifestations’ into the final ranking of the manifestation groups. ‘Other malignancies’ would be more feasible to study in oncology trials, and the pathophysiology of the group ‘Other manifestations’ (with pain and pruritus) was considered not to be not sufficiently understood for the development of a platform trial. All consensus meeting participants completed the third and final questionnaire on the ranking of the eight manifestation groups. Average rankings ranged from 2.3 to 7.5 (Fig. 2). The ‘benign peripheral nerve sheath tumour group’ was deemed most feasible to perform trials for (92% of respondents marking feasibility as ‘easy’), followed by the ‘Cutaneous manifestations’ (83%) and ‘MPNST/Sarcoma’ group (75%). Lowest feasibility was appointed to vascular manifestations (8%) (ANNEX 5).

For NF2, three manifestations were excluded during the consensus meeting, either because effective (surgical) treatments are already available (cataract), or because the manifestations are rare and/or rarely cause significant symptoms (retinal hamartoma, cutaneous schwannoma) (ANNEX 4). Orbital meningioma was grouped under ‘Meningioma’. The remaining manifestations were grouped into a ‘Tumour’ and a ‘Neuropathy’ group, with the first group receiving the highest priority in future platform trials.

For SWN, unilateral vestibular schwannoma and meningioma were excluded because of their rarity and due to the availability of reasonably effective surgical treatment options. Pain was identified as the most important manifestation, given its’ severity and best feasibility for performing platform trials. Loss of function and numbness and/or tingling due to a schwannoma were also considered, but received a lower ranking than pain due to low feasibility for performing platform trials and lack of clear outcome measures.

### Stage 4: Patient representatives’ survey and consensus meeting

The invitation to participate in the survey was sent to 91 patient representatives (ANNEX 6). We obtained 34 responses from patient representatives for NF1, 20 for NF2 and 9 for SWN. All manifestations were assigned high average scores, particularly NF1 manifestations. Since all items were scored in a uniform way, we chose to consider solely the need for new treatment score for our main analysis. The average NT scores for NF1 were highest for the manifestations plexiform neurofibroma (4,0), MPNST (3,9) and spinal nerve root neurofibroma (3,9) (ANNEX 3). Further ranking of the manifestation groups resulted in the highest priority for high grade gliomas (average ranking of 3,2), followed by the groups ‘MPNST/Sarcoma’ (3,4) and ‘benign peripheral nerve sheath tumour’ (4,4) (Fig. 2). Low priority was assigned to vascular manifestations (average ranking 7,3), bone manifestations (7,0) and the developmental/neuropsychological manifestations (6,2). Patient representatives’ rankings differed from those from the NF experts (Fig. 2). Ten manifestations of NF1 displayed a ceiling effect for the NT item, including three manifestations of the ‘benign peripheral nerve sheath tumour’ group, the two sarcoma manifestations (MPNST and other sarcoma) and high grade gliomas.

For NF2, the highest average NT score was appointed to vestibular schwannoma (average score 4,0), but all manifestations within the tumour group received high average scores with relatively small variability in appointed scores (ANNEX 4). The tumour group was assigned the highest average priority ranking of 1,1; for neuropathies this was 2,0 (ANNEX 7). Vestibular schwannoma and ependymoma showed a ceiling effect on NT.

In SWN, pain had highest average scores (ANNEX 4) and also was the only manifestation to show a ceiling effect. Numbness and/or tingling caused by a schwannoma received the lowest average scores. Pain also received the highest average priority ranking of 1,2.

The average rankings of the manifestation groups by the patient representatives were discussed with members from NFPU and CTF during the virtual consensus meeting. The rankings were agreed upon without any changes.

### Stage 5: Final workshop

Based on the results from the NF expert consensus meeting and results from the patient representatives’ survey and consensus meeting, we achieved consensus that for NF1 the following groups will be included into future platform trials: (1) MPNST, (2) benign peripheral nerve sheath tumours, (3) cutaneous manifestations and (4) high grade gliomas. The focus for NF2 will be the tumour group. For SWN, pain will be the top priority.

## Discussion

We performed a five-staged modified Delphi procedure, and were able to reach a consensus on the most important challenges in neurofibromatosis, as seen by experts and patient representatives. We identified four manifestation groups for NF1 that are recommended for future platform trials: MPNST, benign peripheral nerve sheath tumours, cutaneous manifestations and high grade gliomas. For NF2, priority was assigned to the group tumour manifestations (vestibular schwannoma, meningioma and ependymoma). For SWN, pain has been selected as the top priority for future platform trials.

Since platform trials can include multiple conditions in the same trial, either because of similar treatment and/or similar pathophysiology, manifestations could be aggregated into main groups. This allowed for a more extensive selection of manifestations that could be included. A similar strategy can be seen in a newly launched platform trial for NF2 in the USA, where different tumour types are encompassed in the same trial (https://clinicaltrials.gov/ct2/show/NCT04374305). This grouping of manifestations, however, is susceptible to change according to new insights, e.g. the discovery of a new common drug target, or if the pathophysiology of a manifestation can be linked to the pathophysiology of another manifestation (group).

The selection of benign peripheral nerve sheath tumours and MPNST among the NF1 manifestation groups is in alignment with a dominance of plexiform neurofibroma and MPNST in past and current trials in NF1 [4]. Compared to the past couple of years, there is an increase in (planned) trials for oral and/or topical drug treatments and laser/photodynamic therapy for cutaneous manifestations. For NF2, the high priority of vestibular schwannoma and other tumour manifestations agrees with the main focus of current NF2 trials on vestibular schwannoma and meningioma [4].

This study has several strengths. By using modified Delphi questionnaires for the NF experts, we were able to utilise one of the Delphi’s benefits: the involvement of large numbers of participants from all over the world without face-to-face contact [13, 14]. Additionally, the Delphi method avoids the possible dominance of particular individuals by reaching consensus through anonymity and the use of all answers when evaluating the results [15]. The addition of the consensus meeting after the two questionnaires enabled discussion of the results from said questionnaires. Another strength of this study is that we included both the opinion of NF experts and patient representatives in our final selection of manifestations. The inclusion of the patient representatives’ survey into our study has in particular influenced the ranking of HGG (higher ranking) and neurodevelopmental manifestations (lower ranking). Respondents for both the NF expert Delphi and the patient representatives’ survey are also geographically dispersed, limiting country and culture-related bias.

Two main limitations of the NF expert Delphi can be identified. The Delphi method has no standard method for defining consensus. The choice of different exclusion criteria in the first round might have resulted in a different final set of manifestations [14]. Secondly, avoiding the need for face-to-face contact can be advantageous in large international projects like this, the positive aspects of personal interaction are lacking, including discussion of conflicting points and explanation for chosen answers [14]. We provided respondents with the opportunity to give feedback in between questionnaires and arranged the consensus meeting, to deal with these issues.

Partnerships between researchers and patients in the development and performance of clinical trials is increasingly recognised as a priority within the development of new drug therapies. We included the patients’ opinion by performing a patient representatives’ survey. We chose to include patient representatives only, rather than patients themselves, anticipating that they would be able to answer questions for all manifestations of the disease after a specific instruction at the start of the survey. The main limitation of the survey is that patient representatives were asked to score all manifestations separately rather than to consider trade-offs. As such, all manifestations received high average scores without an absolute priority as needed in case of e.g. limited resources in health care, allowing only a small set of manifestations to be studied. Several other limitations exist, one being the relatively small sample size, due to the rarity of the three diseases and our choice to omit patients that were not patient representatives. Another limitation is the convenience sampling method used to select participants. Our sample of patient representatives might not be representative for the NF patient population as a whole. The survey was only offered in English, facilitating English native speakers in particular. This could have caused inclusion bias towards English speaking patient representatives. However, the non-native English speakers (38% of the representatives that completed the survey) did not report significant difficulties with completing the survey in a foreign language in their feedback on the survey. We also did not collect information on level of education, socio-economic status and ethnicity. The impact of socio-economic status may vary strongly amongst countries, and possibly interacts with the psychosocial burden of NF. We did not observe any regional differences between patient representatives from the USA versus European countries, but our small sample size might have influenced this result. There is also no stratification of results for age and patient vs. parent/caregiver respondents of the survey, which could have skewed the results since the NF are progressive conditions. However, results from the patient representatives’ survey were homogenous, suggesting a certain level of data saturation of the results.

Multiple patient representatives reported difficulty in estimating burden and need for treatment for manifestations that they themselves had not experienced. This may have favoured manifestations that had the highest prevalence in our patient representatives’ survey, such as plexiform neurofibroma (68%) and subcutaneous neurofibroma (62%) in NF1 (ANNEX 8). Favouring manifestations may be in particular true for NF2 tumour manifestations (reported prevalence 100% for vestibular schwannoma) compared to NF2 neuropathies (reported prevalence 50% for peripheral neuropathy). In contrast, high grade gliomas were scored very highly, while they were reported prevalent in only 6% of the respondents of our survey. Within the limitations of the results and final ranking of the patient representatives’ survey, we still think that they can serve as an indication of patients’ priorities. Further to our current observations, a new patient directed survey would be of value, including a large group of NF patients with a wide range of manifestations and variation of ethnicity, gender, age and socio-economic status. In order to avoid the ceiling effects seen in our survey, the design of this new survey should include ranking all manifestations individually, to prevent manifestations from being equally scored and prioritised. A Discrete Choice Experiment could also be considered.

The ranking of the high grade gliomas by patient representatives was much higher than the ranking by the NF1 experts in the consensus meeting. NF experts acknowledge the problems with designing a platform trial for high grade gliomas, due to low incidence (risk of dying from a NF1-related malignant brain tumour ranging between a minimum of 3% and a maximum of 9% as calculated from the data of Uusitalo et al. [16]) and short life expectancy from diagnosis [17–19]. This led us to reconsider our decision and to include the high grade gliomas in the final selection. So far, there has been a paucity of research on drug treatments in NF1-related high grade gliomas. The results from this study imply the need for more research for high grade gliomas especially given the low response to current available treatments and lack of early detection methods [20, 21].

Given the high prevalence of neurocognitive manifestations (up to 80% of NF1 children [22, 23]) and the impact on the daily life of patients [24, 25], it is striking that patient representatives appointed relatively low NT scores and priority ranking to this group of manifestations compared to the experts. As a consequence, developmental and neurocognitive manifestations have not been included in our final selection of manifestations for the platform trial. During the consensus meeting, patient representatives suggested that patients are less self-aware of their cognitive disorders and their impact on functioning. Higher awareness of malignancies and the risk they pose may have led to higher prioritisation of malignant manifestations. From a methodological perspective, there is a lack of a standardised set of endpoints for cognitive and behavioural manifestations, and a wide variety of tests and outcome measures have been used in cognitive and behavioural studies in NF1 [26–28]. Lack of a clear set of cognitive and behavioural endpoints hampers the inclusion of this manifestation group into a large scale platform trial. In addition, neurocognitive manifestations require a matched control group to determine the effect of a drug treatment, rather than longitudinal natural history study data, as will be used in EU-PEARL. Although we chose to not include neurodevelopmental manifestations in EU-PEARL based on patient representatives’ priority and the arguments stated above, we acknowledge the importance of clinical trials for this manifestation group.

## Conclusion

In conclusion NF experts and patient representatives consent to prioritise the development of future clinical trials for new drug treatments for MPNST, benign peripheral nerve sheath tumours, cutaneous manifestations and high grade gliomas for NF1; tumour manifestations for NF2; and pain for SWN. The findings of this study are mostly important and relevant to EU-PEARL, to aid the creation of the framework on which the future platform trials can be conducted. This study may serve as a guideline on which manifestation may have highest priority for future research.

## Disclaimer

The authors are member of the EU Patient-centric clinical trial platform (EU-PEARL). EU-PEARL has received funding from the Innovative Medicines Initiative 2 Joint Undertaking under grant agreement no. 853966. This Joint Undertaking receives support from the European Union’s Horizon 2020 research and innovation programme and EFPIA and CTF, Global Alliance for TB Drug Development non-profit organisation, Springworks Therapeutics Inc. This publication reflects the authors’ views. Neither IMI nor the European Union, EFPIA, or any Associated Partners are responsible for any use that may be made of the information contained herein.

## Supplementary information

Supplementary materials: Annex 1, 2, 6, 8, 9 and 10.

Annex 3

Annex 4

Annex 5

Annex 7
