# Supplementary material for: Identifying challenges in neurofibromatosis: a modified Delphi procedure
Source: Eur J Hum Genet. 2021 Apr 26;29(11):1625–33. doi: 10.1038/s41431-021-00892-z (PMC8071842; doi:10.1038/s41431-021-00892-z)
Supplement: Supplementary file 1 — Supplementary materials: Annex 1, 2, 6, 8, 9 and 10. [file 41431_2021_892_MOESM1_ESM.docx]

**Identifying challenges in Neurofibromatosis, a modified Delphi procedure – Supplementary materials: Annex 1, 2, 6, 8, 9 and 10.**

**Table of Contents**

[**ANNEX 1. Likert-scales used in the expert Delphi’s and patient representatives’ survey** 2](#_Toc67568834)

[**ANNEX 2. Manifestation list as used for second questionnaire** 4](#_Toc67568835)

[**ANNEX 6. Characteristics of participants in patient representatives’ survey** 6](#_Toc67568836)

[**ANNEX 8. Patient representatives’ survey: manifestation prevalence in respondents** 7](#_Toc67568837)

[**ANNEX 9. NF experts that participated in the Delphi questionnaires** 8](#_Toc67568838)

[**ANNEX 10. Thematic analysis of the qualitative feedback on the second questionnaire for NF experts – Used as input in expert consensus discussion** 10](#_Toc67568839)

# **ANNEX 1. Likert-scales used in the expert Delphi’s and patient representatives’ survey**

**NF experts – First Delphi questionnaire**

How much priority do you assign to the following manifestations, when trying to create a list of most important manifestations to develop treatment for in future platform trials also known as basket trials?

- 1 = No priority
- 2 = Low priority
- 3 = Moderate priority
- 4 = High priority

**NF experts – Second Delphi questionnaire**

How do you consider the level of need for a new drug treatment for the following manifestations?

- 1 = No need
- 2 = Little need
- 3 = Moderate need
- 4 = Strong need

What known available drug treatments for the following manifestations are you aware of?

- 1 = Aware of easy available treatment, based on strong evidence, part of standard care as a first choice treatment
- 2 = Aware of treatment but lacking strong evidence, not used as first choice treatment
- 3 = Aware of treatment, but only available in experimental setting
- 4 = Not aware of any treatments

How would you judge the level of evidence for these available drug treatments of the following manifestations ?

- 1 = Strong evidence (RCT, systematic review)
- 2 = Moderate evidence (Observational studies)
- 3 = Weak evidence (Case reports, case series)
- 4 = Almost no evidence (Expert opinion)
- 5 = No evidence

**NF patient representatives survey**

How important is it to develop new drug treatment(s) for this manifestation? (On top of already existing treatments, if there are any)

- 1 = Not important
- 2 = Not really important
- 3 = Moderately important
- 4 = Very important

How severe do you consider this manifestation?

- 1 = Not severe at all
- 2 = A little severe
- 3 = Moderately severe
- 4 = Very severe

How much physical burden do you believe that this manifestation generates?

- 1 = No burden
- 2 = Little burden
- 3 = Moderate burden
- 4 = Severe burden

How much psychosocial burden do you believe that this manifestation generates?

- 1 = No burden
- 2 = Little burden
- 3 = Moderate burden
- 4 = Severe burden

How much economic burden do you believe that this manifestation generates?

- 1 = No burden
- 2 = Little burden
- 3 = Moderate burden
- 4 = Severe burden

# **ANNEX 2. Manifestation list as used for second questionnaire**

**Neurofibromatosis type 1**

Neurofibromas

- Cutaneous and subcutaneous neurofibromas
- Plexiform neurofibroma
- Atypical neurofibroma of uncertain malignant potential
- Paravertebral neurofibroma with compression of the spinal cord

Visual

- Optic pathway glioma
- Orbital plexiform neurofibroma

Orthopaedic

- Bone dysplasia - long bones, vertebrae
- Tibial bowing, Pseudarthrosis
- Sphenoid bone dysplasia
- Scoliosis
- Osteoporosis

Neurological and Psychiatric

- Epilepsy
- Migraine
- Cognitive impairment
- ADHD
- Autism
- Emotional and behavioral problems
- Mental health problems (anxiety and depression)
- Problems with speech and language development
- Neurofibromatous neuropathy
- Motor/Coordination problems

Hormonal disorders

- Precocious puberty
- Growth hormone deficiency
- Vitamin D deficiency

Skin

- Pruritus

Malignancies

- MPNST
- Breast cancer in < 50 years
- GIST
- Phaeochromocytoma
- Sarcoma other than MPNST
- Low grade brain glioma
- High grade brain glioma
- Spinal cord glioma
- Other malignancies including lymphoma, leukaemia, bowel cancer, thyroid cancer

Vascular & cardiac disorders

- Renal Artery stenosis
- Cerebrovascular – including moya moya syndrome, aneurysm, haemorrhage, occlusion and fistula
- Hypertension

Other

- Sleep disorder
- Fatigue/strength
- Pain

**Neurofibromatosis type 2**

- Vestibular schwannomas
- Schwannomas in other location
- Meningioma
- Ependymoma
- Cutaneous or subcutaneous schwannomas
- Visual complications caused by cataract
- Visual complications caused by orbital meningiomas
- Visual complications caused by retinal hamartomas
- Peripheral neuropathy/Pain
- Mononeuropathy

**Schwannomatosis**

- Chronic pain caused by a schwannoma
- Impaired or loss of function due to a schwannoma
- Numbness or tingling due to a schwannoma
- Meningioma
- Vestibular schwannoma

# **ANNEX 6. Characteristics of participants in patient representatives’ survey**

*Details on the number of invitations sent and number of received responses per patient organisation for the patient representatives’ survey.
NF1 = Neurofibromatosis type I, NF2 = Neurofibromatosis type II, SWN = Schwannomatosis, CTF = Children’s Tumor Foundation (USA), NFPU = NF Patients United (Europe).*

|  | **CTF** | | **NFPU** | | **Other patient organisations** |
| --- | --- | --- | --- | --- | --- |
|  | **Invitations sent** | **Responses** | **Invitations sent** | **Responses** | **Responses** |
| **NF1** | 30 | 18 | 25 | 9 | 7 |
| **NF2** | 10 | 6 | 15 | 13 | 2 |
| **SWN** | 6 | 3 | 5 | 4 | 2 |

*Characteristics of the respondents of the patient representatives’ survey.
NF1 = Neurofibromatosis type I, NF2 = Neurofibromatosis type II, SWN = Schwannomatosis.*

|  |  | **Age** | | | | **Region** | | **Role** | | |
| --- | --- | --- | --- | --- | --- | --- | --- | --- | --- | --- |
|  | **Number of respondents** | **11 – 20 years** | **21 – 40 years** | **41 – 65 years** | **66+ years** | **Europe** | **USA** | **Patient** | **Parent/caregiver** | **Other** |
| **NF1** | 34 | 1 (3%) | 12 (35%) | 18 (53%) | 3 (9%) | 14 (41%) | 20 (59%) | 12 (36%) | 18 (53%) | 4 (12%) |
| **NF2** | 20 | 2 (10%) | 12 (60%) | 6 (30%) | 0 (0%) | 14 (70%) | 6 (30%) | 13 (65%) | 4 (20%) | 3 (15%) |
| **SWN** | 9 | 1 (11%) | 1 (11%) | 7 (78%) | 0 (0%) | 5 (56%) | 4 (44%) | 6 (67%) | 1 (11%) | 2 (22%) |

# **ANNEX 8. Patient representatives’ survey: manifestation prevalence in respondents**

*Characteristics of the respondents of the patient representatives’ survey: the number of respondents that have the manifestation, (or the patient they represent has this manifestation) followed by the percentage.*

|  | Yes (%) | No (%) |
| --- | --- | --- |
| **NF1** |  |  |
| **Benign peripheral nerve sheath tumours** |  |  |
| Plexiform neurofibroma | 23 (67,6) | 11 (32,4) |
| Subcutaneous neurofibroma | 21 (61,8) | 13 (38,2) |
| Neurofibroma near the spinal cord (spine root neurofibroma) | 8 (23,5) | 26 (76,5) |
| **MPNST/Sarcomas** |  |  |
| Malignant peripheral nerve sheath tumour | 4 (11,8) | 30 (88,2) |
| Sarcoma (other than MPNST) | 1 (2,9) | 33 (97,1) |
| **Cutaneous manifestations** |  |  |
| Cutaneous neurofibroma | 20 (58,8) | 14 (41,2) |
| **Developmental / neuropsychological manifestations** |  |  |
| Problems with coordination and/or motor skills | 20 (58,8) | 14 (41,2) |
| Emotional and/or behavioural problems | 15 (44,1) | 19 (55,9) |
| Cognitive impairment | 14 (41,2) | 20 (58,8) |
| Problems with speech and language development | 12 (35,3) | 22 (64,7) |
| ADHD | 10 (29,4) | 24 (70,6) |
| Autism spectrum disorder | 5 (14,7) | 29 (85,3) |
| **High grade glioma** |  |  |
| High grade brain glioma | 2 (5,9) | 32 (94,1) |
| **Low grade glioma** |  |  |
| Low grade brain glioma | 9 (26,5) | 25 (73,5) |
| Optical pathway glioma | 9 (26,5) | 25 (73,5) |
| Low grade glioma in the spinal cord | 1 (2,9) | 33 (97,1) |
| **Bone manifestations** |  |  |
| Tibial bowing / pseudoarthrosis | 6 (17,6) | 28 (82,4) |
| Scoliosis | 11 (32,4) | 23 (67,6) |
| Bone dysplasia of the long bones and/or vertebrae | 4 (11,8) | 30 (88,2) |
| Sphenoid bone dysplasia | 3 (8,8) | 31 (91,2) |
| Osteoporosis | 2 (5,9) | 32 (94,1) |
| **Vascular manifestations** |  |  |
| Cerebrovascular manifestations | 3 (8,8) | 31 (91,2) |
| Renal artery stenosis | 2 (5,9) | 32 (94,1) |
| **Other malignancies** |  |  |
| Breast cancer | 2 (5,9) | 32 (94,1) |
| Phaeochromocytoma | 2 (5,9) | 32 (94,1) |
| Gastrointestinal stromal tumour | 1 (2,9) | 33 (97,1) |
| Other malignancies | 1 (2,9) | 33 (97,1) |
| **Other manifestations** |  |  |
| Pain | 16 (47,1) | 18 (52,9) |
| Pruritus | 12 (35,3) | 22 (64,7) |
| **NF2** |  |  |
| **Tumours** |  |  |
| Vestibular schwannoma | 20 (100) | 0 (0) |
| Schwannoma in other location | 16 (80) | 4 (20) |
| Meningioma | 14 (70) | 6 (30) |
| Ependymoma | 6 (30) | 14 (70) |
| **Neuropathies** |  |  |
| Peripheral neuropathy | 10 (50) | 10 (50) |
| Mononeuropathy | 3 (15) | 17 (85) |
| **Excluded** |  |  |
| Cutaneous or subcutaneous schwannoma | 11 (55) | 9 (45) |
| Visual complication caused by cataract | 11 (55) | 9 (45) |
| Visual complications caused by retinal hamartoma | 5 (25) | 15 (75) |
| **Schwannomatosis** |  |  |
| Pain | 7 (77,8) | 2 (22,2) |
| Numbness or tingling due to schwannoma | 7 (77,8) | 2 (22,2) |
| Impaired or loss of function due to schwannoma | 6 (66,7) | 3 (33,3) |

# **ANNEX 9. NF experts that participated in the Delphi questionnaires**

Only the NF experts that agreed to their name being published are listed here, so this list does not feature all participants.

**CTF-Europe CCAB**

A. A. Azizi, MD – Department of Pediatrics and Adolescent Medicine, Division of Neonatology, Pediatric Intensive Care and Neuropediatrics, Medical University of Vienna, Austria

A. Bakker, PHD – President of Children’s Tumor Foundation, New York, USA

I. Blanco, MD – Department of Clinical Genetics, Hospital Germans Trias i Pujol, Badalona, Spain

G. Evans, MD - Centre for Genomic Medicine, Division of Evolution and Genomic Sciences, University of

Manchester, St Mary's Hospital, Manchester, UK

R. E. Ferner, MD - Department of Neurology, Guy’s and St. Thomas’ NHS Foundation Trust London, UK

M. Kalamarides, MD PhD – Department of Neurosurgery, Hôpital Pitié-Salpêtrière-APHP, Sorbonne université, Paris, France

E. Legius, MD – Department of Clinical Genetics, UZ Leuven, Belgium

V. Mautner, MD – Department of Neurology, University Medical Center Hamburg-Eppendorf, Germany

R. Oostenbrink, MD – Department of Pediatrics, Sophia’s Children’s Hospital, Rotterdam, The Netherlands

S. R. Plotkin, MD - Department of Neurology and Cancer Center, Massachusetts General Hospital, Boston, MA, USA

C. Potratz, MD – Department of Pediatric Neurology, Charité Universitätsmedizin Berlin, Germany

C. F. Rustad – Department of Medical Genetics, Oslo University Hospital, Oslo, Norway

E. Trevisson, MD, PhD – Clinical Genetics Unit, Dept. of Women's and Children's Health

University of Padova, Padova, Italy

P. Wolkenstein, MD – Department of Dermatology, Hôpital Universitaire Pitié-Salpêtrière (APHP), Paris, France

**ERN GENTURIS**

I. Blanco, MD – Department of Clinical Genetics, Hospital Germans Trias i Pujol, Badalona, Spain

C. Cassiman, MD, PhD – Department of Ophthalmology, UZ Leuven, Leuven, Belgium

G. Evans, MD - Centre for Genomic Medicine, Division of Evolution and Genomic Sciences, University of

Manchester, St Mary's Hospital, Manchester, UK

R. E. Ferner, MD - Department of Neurology, Guy’s and St. Thomas’ NHS Foundation Trust London, UK

E. Legius, MD – Department of Clinical Genetics, UZ Leuven, Belgium

R. Oostenbrink, MD – Department of Pediatrics, Sophia’s Children’s Hospital, Rotterdam, The Netherlands

H. Salvador, MD – Department of Oncology and Haematology, Sant Joan de Deu Barcelona Children’s Hospital, Barcelona, Spain

K. Wimmer, PhD – Institute of Human Genetics, Medical University of Innsbruck, Innsbruck, Austria

P. Wolkenstein, MD – Department of Dermatology, Hôpital Universitaire Pitié-Salpêtrière (APHP), Paris, France

**Other**

M. J. Fisher, MD – Division of Oncology, The Children’s Hospital of Philadelphia, USA

D. Halliday, MD, Oxford Centre for Genomic Medicine, Oxford University Hospitals NHS Trust, Oxford, UK

J. T. Jordan, MD, MPH - Pappas Center for Neuro-Oncology, Massachusetts General Hospital, Boston, USA

M. Karajannis, MD, MS – Department of Pediatrics, Memorial Sloan Kettering Cancer Center, New York, USA

H. Kehrer-Sawatzki, PhD – Institute of Human Genetics, University of Ulm, Ulm, Germany

M. Larralde, MD – Department of Dermatology, Hospital Alemán, Buenos Aires, Argentina

R. Listernick, MD – Department of Pediatrics, Ann & Robert H. Lurie Children's Hospital of Chicago, USA

V. Merker, PHD – Department of Neurology, Massachusetts General Hospital, USA

C. Moertel, MD – Department of Pediatrics, University of Minnesota Medical School, Minneapolis, USA

J. Ngeow Yuen Yie, MD – Cancer Genetics service, National cancer Centre Singapore, Singapore

R. J. Packer, MD – Center for Neuroscience and Behavioral Medicine, Children’s National Hospital, Washington D.C., USA

L. Papi, MD – Department of Biomedical Experimental and Clinical Sciences, University of Florence, Italy

T. Rosser, MD – Department of Neurology, Children’s Hospital Los Angeles, USA

M. J. Smith, PHD - Centre for Genomic Medicine, Division of Evolution and Genomic Sciences, University of Manchester, St Mary's Hospital, Manchester, UK

A. Varan, MD - Department of Pediatric Oncology, Hacettepe University Faculty of Medicine, Ankara, Turkey

# **ANNEX 10. Thematic analysis of the qualitative feedback on the second questionnaire for NF experts – Used as input in expert consensus discussion**

**The thematic analysis**

A thematic analysis was performed on the free text comments that we received on the second round of the Delphi. First the comments were coded manually, followed by aggregating the codes into themes. We identified three major themes: Treatment, Expertise and Phrasing. As the majority of comments were in the Treatment theme, we distinguished four subthemes:

- Treatment available, but with limited effect
- Treatment available, but not specific for the condition NF
- Treatment available, but no drug treatment (alternative treatment)
- Availability of drugs in routine care

**Summary of comments:**

**Treatment**

*Issues on treatment available, but with limited effect*

There are manifestations where treatment is available, but the effect is limited. This was reported for the following manifestations:

NF1: Vitamin D deficiency, high grade glioma, MPNST, optic pathway glioma

NF2: Ependymoma, pain/neuropathy, vestibular schwanomma

*Treatment available, but not specific for the condition NF*

Some manifestations also appear in the general population, and treatments are available, but it’s not clear if these treatments have the same effect on NF patients. This was reported for the following manifestations:

NF1: Pain relief, sleep disorder, low grade glioma, MPNST, fatigue, growth hormone deficiency

NF2: Meningioma, ependymoma, pain

*Treatment available, but no drug treatment (alternative treatment)*

For some manifestations there are effective treatments available that are no drug treatments, so there is no / less need for a new drug treatment. This was reported for the following manifestations:

NF1: Renal artery stenosis, phaeochromocytoma

*Availability of drugs in routine care*

Some treatments are only available in an experimental setting. This was reported for the following manifestations:

NF2: Plexiform neurofibroma (Selumetinib)

**Phrasing**

Most comments were related to interpretation of questions on treatment, which have been included in the Treatment theme.

An example of a specific comment on item phrasing:

“The question peripheral neuropathy/pain is confusing. I presume you mean generalised axonal peripheral neuropathy”

“Whereas in low grade glioma: why do you separate optic pathway from e.g. hypothalamus glioma”

**Expertise**

For NF1, one respondent reported no expertise, and for NF2 and SWN this was four respondents. These respondents were excluded from the results for the disease for which they had no expertise. Two respondents reported subspecialist expertise in NF1, but were included into the results.

**Examples of responses**

“there are proven treatments for vestibular schwannoma, but helpful in only a minority of patients”

“It is difficult to determine whether known treatments for disorder in the general public are equally effective for NF e.g. pain relief, sleep disorder”

**“**please make note that for several of the manifestations presented, surgery is appropriate and complete treatment (i.e. renal artery stenosis and pheochromocytoma). In such cases a drug does not exist, but is not needed.”

**Raw comment data**

**NF1**

1. It is difficult to determine whether known treatments for disorder in the general public are equally effective for NF e.g. pain relief, sleep disorder
2. Not expert in NF1
3. I am an ophthalmologist, please consider glioma questions as most relevant for me
4. Some of the diseases, symptoms mentioned are well described in nonNF1 population, but the incidence, the proper management in NF1 are not. I.e treatment of Low grade glioma in NF1 probably should be different to sporadic ones. Or scholiosis, fatigue, MPNST... SInce the biology is different, the treatment could be or should be different
5. For tumors sarcoma, glioma chemotx (drug) is only part of treatment, wih quite evidence but the complete treatment (surgery, radiotx and chemotx) together has limited result, so need is high. cognitive impairment/emotional: there is evidence for some drugs that it doesnot work. vit D: question is whether tx has effect. Growth hormone def: we lack evidence if it works in NF specifically. renal artery stenosis: we have treatment for hypertension, but not for the stenosis itself
6. autism should be replaced with social skills; surgery works well for a number of the manifestations outlined above meaning medications really not needed.
7. please make note that for several of the manifestations presented, surgery is appropriate and complete treatment (i.e. renal artery stenosis and pheochromocytoma). In such cases a drug does not exist, but is not needed.
8. Problem in the formulation. No drug treatment but surgical treatment
9. Thank you for providing this survey and for all your efforts! But I fear that the questions are not leading to the right answers. The questions should rather have been: are you aware of an EFFECTIVE treatment for... Because I am e.g. aware of multiple treatments for high grade glioma, but their effectiveness is more than limited. The same holds true for MPNST. State of the art oncological treatment is - at least in paediatrics - always performed in clinical trials. Therefore such a therapy will / should only be available in an experimental setting. It is therefore hard to answer in the way the question was probably intended. Selumetinib has not yet been marketed and is therefore only available in clinical trials (or named patient use). Therefore e.g. for plexiform NF my answer whether novel treatment was needed is yes - after marketing of the drug my answer would be no... Whereas in low grade glioma: why do you separate optic pathway from e.g. hypothalamus glioma? (two different parts of the survey) The treatments are the same and effectiveness is proven for tumour control. Concerning tumour control I would therefore feel it to be unnecessary to develop new drug treatments. BUT: regarding vision there is no evidence of effectiveness of the standard of care treatment. Therefore novel drugs are urgently needed for this aspect. (this is why I've answered: there is a clear standard of care, but answered the next question with a clear need for new drugs - thus the answers you may get in your survey may be conflicting). For other symptoms / manifestations there is a clear need for new treatment options, but I fear that e.g. a drug treatment for scoliosis (due to the different reasons in NF1 patients) or sphenoid dysplasia is in my opinion clearly not a feasible option. This is the area of surgery - but if someone came up with a drug, I would definitely offer it to my patients. I just fear that money and efforts should better focus on other aspects with a higher likelihood of success.
10. I would have inserted also a column with "Aware of available treatment used for other diseases"

**NF2**

1. Again I am assuming surgery and radiation are not included as 'treatments' (**This was before rephrasing of questions)**
2. The question peripheral neuropathy/pain is confusing. I presume you mean generalised axonal peripheral neuropathy. Not all generalised neuropathy causes pain. I do not understand this question
3. Same as NF1. Although there are a lot of information and review for some of the symptoms or conditions mentioned above (meningioma,ependimoma,pain...). The biology in NF2 is very different than on sporadic cases, so real evidence in this conditions is much lower
4. treatment ependymoma is only as adjuvans and effect limited. treatment for pain/neuropathy is there with evidence, but effect is limited, indicating need. Do not know on evidence is partly due to there is no study
5. there are proven treatments for vestibular schwannoma, but helpful in only a minority of patients and time limited
6. I am not NF2 specialist
7. please only consider ophthalmological questions answers for me
8. I have only little experience in NF2
9. I am dermatologist (no nf2 experience)

**SWN**

1. some limited drug treatment for schwannomotosis extrapolated from knowledge of NF2, but lacking evidence if it works in schwannomatosis as well
2. I am not schwannomatosis specialist
3. I have no experience in Schwannomatosis
4. i am dermatologist (no schwannomatosis experience)
